# Pro-Opiomelanocortin and Melanocortin Receptor 3 and 4 Mutations in Genetic Obesity

**DOI:** 10.3390/biom15020209

**Published:** 2025-02-01

**Authors:** Tulin Yanik, Seyda Tugce Durhan

**Affiliations:** 1 Department of Biological Sciences, Middle East Technical University, Ankara 06800, Türkiye; 2 Department of Biochemistry, Middle East Technical University, Ankara 06800, Türkiye; e207195@metu.edu.tr

**Keywords:** pro-opiomelanocortin, melanocortin receptors, genetic obesity

## Abstract

Genetic obesity results from loss-of-function mutations, including those affecting the leptin–melanocortin system, which regulates body weight. Pro-opiomelanocortin (POMC)-derived neurohormones act as ligands for melanocortin receptors (MCRs), regulating the leptin–melanocortin pathway through protein–protein interactions. Loss-of-function mutations in the genes encoding POMC, MC3R, and MC4R can lead to the dysregulation of energy expenditure and feeding balance, early-onset obesity, and developmental dysregulation. Recent studies have identified new genetic regulatory mechanisms and potential biomarker regions for the *POMC* gene and MC4R secondary messenger pathway associated with obesity. Recent advances in crystal structure studies have enhanced our understanding of the protein interactions in this pathway. This narrative review focuses on recent developments in two key areas related to POMC regulation and the leptin–melanocortin pathway: (1) genetic variations in and functions of POMC, and (2) MC3R and MC4R variants that lead to genetic obesity in humans. Understanding these novel mutations in POMC and MC4R/MC3R, as well as their structural and intracellular mechanisms, may help identify strategies for the treatment and diagnosis of obesity, particularly childhood obesity.

## 1. Introduction

Genetic and environmental factors influence the development of obesity, which can be categorized as monogenic, syndromic, or common obesity. Monogenic and syndromic obesity occur due to loss-of-function mutations in a single gene or multiple genes, respectively, and demonstrate a Mendelian pattern of inheritance. On the other hand, a combination of genetic and environmental factors results in common obesity. The World Health Organization (WHO) defines individuals with a body mass index (BMI) ≥ 30 as obese. A recent study published in *The Lancet* [1] indicates that more than one billion people are obese, and obesity is remarkably prevalent among children and adolescents aged 5 to 19 years [1,2]. Considering that obesity is associated with a high risk of cardiovascular disease, type 2 diabetes, a shorter life expectancy, and poor quality of life, the increasing prevalence of obesity underlines the necessity of obesity research and the development of effective therapeutic approaches.

Many biological factors and hormones regulate body weight. The hypothalamus, a key part of the central nervous system (CNS), controls energy metabolism in response to food intake through the melanocortin pathways, a process further mediated by brain–gut nutrient signaling [3,4,5]. In the arcuate nucleus (ARC) of the hypothalamus, the leptin–melanocortin pathway regulates energy metabolism and appetite through neuropeptide Y (NPY), agouti-related protein (AgRP), pro-opiomelanocortin (POMC), and cocaine- and amphetamine-related transcript (CART), which show altered expression in response to obesity [3,6]. These pathways are modulated by leptin and insulin, which negatively affect the expression of NPY and AgRP and positively affect POMC and CART expression [7]. In many cases, loss-of-function mutations in the genes involved in these pathways are a hallmark of severe childhood obesity [8].

**Figure 1:**
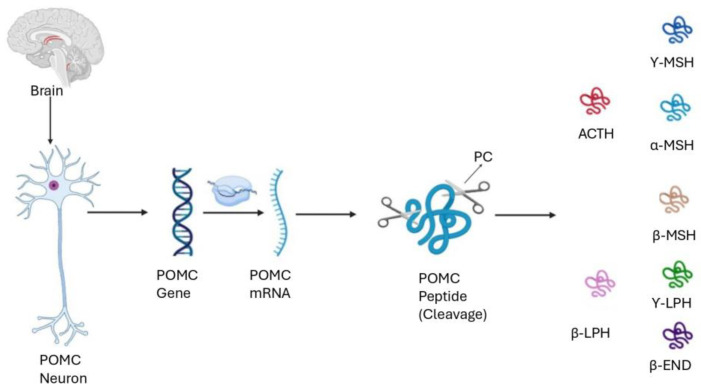
Post-translational cleavage of POMC. Neuropeptides derived from POMC are produced in secretory vesicles via the action of prohormone convertase (PC) enzymes. ACTH—adrenocorticotropic hormone; α-MSH—α-melanocyte-stimulating hormone; β-MSH—β-melanocyte-stimulating hormone; γ-MSH—γ-melanocyte-stimulating hormone; γ-LPH—γ-lipotropic hormone; β-LPH—β-lipotropic hormone; β-END—β-endorphin (modified from Pritchard and White [15]).

The 31 kDa human POMC protein is a prohormone that is processed to produce eight physiologically active neurohormones: β-endorphin, alpha-melanocyte-stimulating hormone (α-MSH), β-MSH, γ1-MSH, γ3-MSH, beta-lipotropin hormone (β-LPH), γ-LPH, and adrenocorticotropic hormone (ACTH) (Figure 1) [9]. The neurohormones derived from POMC are important for the regulation of various physiological processes such as immunological regulation, energy balance, and stress responses [10,11,12]. POMC and POMC-derived peptides are also secreted from peripheral tissues, such as skin, and in the traditional hypothalamic–pituitary–adrenal axis that controls stress responses, demonstrating their significant and broad influence on physiological processes [12,13,14,15].

Five distinct melanocortin receptors mediate the POMC-derived peptides’ unique functions in various organs. One of the first cleavage products of cleaved POMC is ACTH, which comprises 39 amino acids and is capable of stimulating all melanocortin receptors (MCRs). Stress stimulates the production of ACTH, which is secreted from the anterior pituitary and is believed to primarily moderate the adrenocortical response during stress. Upon reaching the adrenal gland, ACTH stimulates the synthesis of glucocorticoids in the adrenal gland cortex and promotes melanogenesis in the skin [16]. High serum levels of ACTH have been associated with elevated melanogenesis in some patients [17]. ACTH also plays a role in the synthesis and release of adrenal cortisol and androgens via the coupling of its receptor, MC2R [18,19,20]. Recent studies have also linked ACTH levels with polycystic ovary syndrome (PCOS) in females [21,22]. Another product of POMC, α-MSH, is generated in the skin and has anti-inflammatory and immunomodulatory effects. It regulates pigmentation by binding to MC1R and MC2R and induces melanogenesis in human melanocytes [23]. More importantly, and perhaps most crucially, α-MSH produced in the hypothalamus is the primary neurohormone regulating appetite and enhancing energy expenditure by binding to MC3R and MC4R [15,24]. Another neurohormone derived from POMC, β-LPH, is regarded as a potential aldosterone-stimulating agent and is involved in lipid mobilization [25]. However, recent research has demonstrated that its main function is as a precursor to endorphin and MSH hormones. Since humans lack the cleavage sites for prohormone convertase (PC) 2, LPH is not processed further into γ-LPH and β-endorphin [26,27]. Another product of POMC, β-MSH, consists of 22 amino acids and binds to MC4Rs with high affinity in vitro. Although not as effective as α-MSH, β-MSH participates in the regulation of body weight in humans through stimulating hypothalamic receptors [28]. β-endorphin, yet another product of POMC, binds to α-opioid receptors but lacks the tetrapeptide sequence shared by the other melanocortins. It is also involved in pain perception and the analgesic reaction [29]. Finally, γ-MSH plays a dynamic role in controlling metabolism due to its influence on neuropeptide systems in the ARC. Similarly to the binding of α-MSH to MC4R, γ-MSH binds to MC3R to regulate appetite and energy expenditure, and a loss of function of both receptors leads to severe early-onset obesity [30,31,32]. The importance of MC3R in the fasting response was highlighted by the increased refeeding that is observed following fasting, and its coupling with γ-MSH was found to increase appetite [24,33].

The observed phenotypic differences between MC3R and MC4R in the regulation of the leptin–melanocortin pathway could be due to different protein–protein interactions and ligands [34,35,36]. According to Müller et al. [37], E3 ligase protein ring finger 11 (RNF11) can physically and simultaneously interact with and regulate both the MC3R and MC4R secondary messenger pathways. Although MC3R and MC4R exhibited similar total expression levels, their cellular membrane surface expression patterns differed when they were co-expressed with RNF11 E3 ligase [37]. This suggests that a regulatory difference exists between these receptors, likely due to downregulation or sequestration. Interestingly, Wellman et al. [38] demonstrated that growth hormone secretagogue receptor 1A (GHSR1A), which plays a significant role in the regulation of stress, feeding, and the reward mechanism, can form a heterodimer with MC3R, enhancing the G_αs_-activated secondary messenger pathway while inhibiting GHSR1A interaction with MC3R-specific ligands [38].

**Figure 2:**
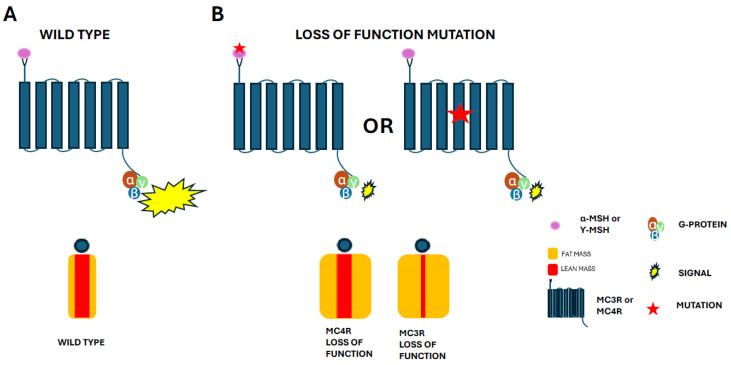
Schematic representation of POMC-derived peptide ligands and MC3R/MC4R signaling mechanisms in body weight regulation. (**A**) Interactions between WT POMC-derived peptides and MCR lead to conformational changes, G-protein signaling, and production/activation of secondary messenger molecules. (**B**) Mutations in POMC-derived ligands or MCRs may induce a loss of function. Alterations in receptor structure and MCR–ligand interactions may prevent the desired conformational shift; therefore, the generation of secondary messenger signal molecules is poor or non-existent due to reduced G-protein signaling.

POMC deficiency mutations are often associated with obesity, non-alcoholic fatty liver disease, type 2 diabetes, and certain circulatory problems; in particular, the chronic activation of POMC neurons reduces blood pressure [39]. Aside from the loss of function of POMC itself, loss-of-function mutations in MC3R and MC4R also cause dysregulation of POMC-related pathways, leading to severe childhood obesity (Figure 2) [40,41]. This review highlights the recent findings on novel mutations of POMC, its derived obesity-related hormones, and their interactions with MCRs. It also focuses on the developments in our understanding of the regulation of MC4R and MC3R through variants identified in a population of obese children. The objective is to outline their signaling pathways to further our understanding and management of genetically induced early-onset childhood obesity.

## 2. POMC Mutations

The ineffective processing of POMC in the pituitary gland results in a markedly increased quantity of unprocessed POMC in the circulation. The ratio of unprocessed POMC is frequently used to evaluate the functions of POMC-derived neuropeptides [15,42]. Genetic variants have demonstrated the key role of POMC in various physiological processes [43,44]. Changes in DNA methylation patterns in the promoter region of the *POMC* gene may affect *POMC* expression, and these modifications could alter glucose homeostasis, energy expenditure, and food intake regulation. Other factors such as prenatal alcohol exposure, stress, a high-fat diet (HFD), psychotropic drugs, and malnutrition have also been linked to epigenetic modifications in the *POMC* gene that may cause metabolic problems [43,45,46,47].

Various mutations in the *POMC* gene have been reported. Some of these mutations, which are associated with severe monogenic and syndromic obesity, might also be linked to other genetic syndromes such as Cushing syndrome, which is characterized by chronically increased cortisol levels and weight gain [48]. Araki et al. [49] studied samples obtained from patients with Cushing syndrome in order to investigate the 5′ ends of the *POMC* mRNA region, specifically between +6331 and +7120 bp downstream of the previously identified *POMC* transcription start site. After sequencing the transcription region, they discovered a novel second promoter region. The discovery was made using the luciferase reporter assay and verified via pull-down assays. Interestingly, the second promoter region was found to be more active in Cushing-related mutant samples compared to wild-type (WT) cells. The second promoter was discovered to be an enhancer for the first promoter, and the demethylation of the second promoter may play a role in silencing the *POMC* gene [49]. The second promoter was found to be located next to the CREB and STAT3/5 binding sites and is sequentially controlled by these factors. Unfortunately, the lack of information on the body weight of the patients made it difficult to determine whether there is a relationship between the regulation of the second *POMC* promoter and body weight control mechanisms.

DNA motifs are short, repetitive patterns of nucleotides within a DNA sequence and have a variety of biological functions [50]. KKRRP is a functional motif located in the *POMC* gene that is thought to be necessary to bind MC2R [51]. Van der Valk et al. [52] discovered a novel variant in the KKRRP motif in the part of the *POMC* gene that encodes ACTH, resulting in KRR-ACTH. Because this was the first variant detected at the PC2 cleavage site, its implications are crucial to understanding POMC regulation in humans. Compared to the WT, the mutant ACTH neurohormone was found to be less potent based on the reduced activity of the cAMP secondary messenger. In vitro studies showed that the KRR-ACTH variant exhibited a reduced capacity to activate MC2R. The results of this study demonstrated that ACTH plays a significant role in hypocortisolism and feeding control mechanisms and that mutations in the PC2-cleavage region alter α-MSH production [52].

Non-alcoholic fatty liver disease (NAFLD) is characterized by an accumulation of fat in the liver that is not due to alcohol consumption. NAFLD is associated with type 2 diabetes and is often related to obesity [53], indicating a relationship between mutations in obesity-related genes and NAFLD. In 18% of patients suffering from NAFLD, partial or complete deletions were found in the genes encoding POMC and leptin receptor [54]. It is important to understand the regulation of POMC and its link to obesity from a biochemical perspective to clearly describe its function. In this context, it is hypothesized that *POMC* gene mutations can cause an increase in neutral triglyceride lipid droplets within hepatocytes. This accumulation may lead to an elevated amount of free fatty acids (FFAs) and a decrease in FFA beta-oxidation in the liver, which are associated with obesity. Although this study did not further investigate treatment strategies, hormone replacement therapy might aid in patient recovery [55]. Investigating the regulation of the *POMC* gene by obesity-associated genes is a hotspot in obesity research. In addition, Plum et al. [56] demonstrated that the deletion of the *FOXO* gene in POMC neurons increased the carboxy-peptidase E-dependent processing of POMC into α-MSH, which exerted an appetite-decreasing effect [56].

## 3. MC3R and MC4R Mutations

MCRs belong to the G-protein-coupled receptor (GPCR) family and are expressed in various tissues, including the hypothalamus. POMC-derived α-, β-, and γ-MSH serve as ligands for MC3R and MC4R, modulating appetite and energy expenditure. POMC mutations may affect POMC-derived peptide products. For example, one study found that β-MSH and β-endorphin were not being produced because of a shared POMC mutation; however, α-MSH production was observed (both humans and dogs generate α-MSH and β-MSH from the POMC propeptide, while rodents only produce α-MSH) [57]. MC3R and MC4R loss-of-function mutations have been shown to disrupt the leptin–melanocortin pathway and POMC-related signaling, triggering metabolic dysregulation (Figure 2). Increased food intake, reduced energy expenditure, and increased body fat were noted in mouse models of MC4R-related obesity. A loss-of-function mutation in MC3R resulted in an increased fat mass, decreased lean mass, and no difference in food consumption in subjects fed an HFD compared to a control group. Interestingly, no changes in energy expenditure and food consumption occurred with a chow diet, although there was an increase in body weight [24,46,58,59,60]. During fasting, MC3R loss-of-function mutations caused faster weight loss and slower weight gain during the refeeding period. Although MC4R-related obesity is well documented, MC3R-related obesity is not as well studied. Population studies have shown, however, that MC3R-related genetic obesity is widespread in obese adolescent populations. These include the MC3R loss-of-function mutation Thr6Lys/Val81Ile, which accounts for 8% of childhood obesity cases [31]. The consequences of MC3R mutations include severe obesity and ultradian rhythm disruptions [41,61]. One study, using a mature female *Mc3r*-null mouse model, identified a rare human loss-of-function mutation in MC3R that resulted in obesity, a decreased lean mass, and increased ovulation length [41].

The relationship between MC3R and the circadian rhythm is debated in the literature on MC3R variants. A patient with the unusual loss-of-function variant MC3R G240W demonstrated early-onset obesity and a late onset of puberty. This variant also caused decreased cAMP signaling activity compared to WT MC3R in vitro. In vivo studies also found that the loss of function of MC3R affects sexual maturation and the ultradian cycle [31,41]. Interestingly, Ong et al. [62] identified three novel mutations in MC3R and MC4R. These mutations, MC3R c.151G > C (p.Val51Leu), MC4R c.127C > A (p.Gln43Lys), and MC4R c.272T > G (p.Met91Arg), were predicted to cause a delay in puberty in humans. However, because of their young ages, all male and female probands were identified in the prepubertal stage [62]. It was discovered that MC3R Glu43Lys and Ile146Asn did not respond to α-MSH, whereas Ala33Thr and Val51Leu were functional. The Val51Leu variant was present in an obese patient in combination with Glu43Lys, which was also determined to be nonresponsive to α-MSH. Despite responding to α-MSH, the Ala33Thr variant was associated with the obese phenotype. The differences in the phenotypes associated with different loss-of-function variants of MC3R could be due to factors such as different protein–protein interactions or conformational changes in the receptor.

Previously, it was thought that MC3R’s role in the leptin–melanocortin pathway was redundant. However, the current research and findings on MC3R protein–protein interactions suggest otherwise, highlighting the significance of MC3R in this system [63]. Studies have demonstrated that the dimerization of MCRs, specifically MC3R with GHSR1A, increased MC3R-related cAMP signaling [64]. This dimerization might be involved in tuning MC3R signaling and influencing both the leptin–melanocortin pathway and developmental pathways [65]. Thus, the structural characteristics of these protein interactions are crucial to both understanding the molecular mechanisms of MC3R and developing treatment strategies for MC3R-related obesity. Recently, Feng et al. [66] released the first model of the MC3R crystal structure, complementing the findings from computational structural studies and supporting the research on MC3R–protein interactions [66]. Understanding the structural mechanisms and dynamics of the protein interactions involving MCRs is critical to explaining their influence on energy expenditure and food intake mechanisms. MC4R is the most studied MCR, and mutations in its gene are common causes of monogenic obesity. Loss-of-function mutations of MC4R result in increased food intake and decreased energy expenditure, as well as severe early-onset obesity in many human populations. Studies have shown that the phenotypic features of the loss of function of MC4R in children are increased fat and lean masses, increased linear growth, increased bone mineral density, hyperphagia, and hyperinsulinemia [67]. Obesity has been associated with MC4R loss-of-function mutations through the malfunction of the G_αs_-activated cAMP secondary messenger pathway [68]. This association is supported by the work of Heyder et al. [69], who published a crystal structure of MC4R coupled with G_αs_ in the ligand-bound active state, which has broadened the scope of structural and computational studies on MC4R protein interactions [69]. Metzger et al. [34] linked the MC4R F51L loss-of-function variant, which could still couple to Gαs and elevate cAMP levels, to obesity. However, the activation of G_αq/11_ by MC4R F51L was reduced by α-MSH stimulation, which did not occur with the WT. Interestingly, this study indicated that the control of food intake by MC4R was similarly dependent on G_αq/11_. Blocking G_αq/11_ signaling prevented MC4R from inhibiting food intake and it reduced the inositol phosphate 1 (IP1) response [34]. The novel mutation MC4R Gln43Lys exhibited decreased functional signaling, although it could be mitigated by an increase in ligand concentration. On the contrary, MC4R Met91Arg demonstrated a complete loss of function. These striking findings on the coupling of MC4R with G_q/11_α and its effect on the IP1 response create new opportunities for research on MCRs related to obesity and signaling regulation that may lead to new therapeutic approaches.

## 4. Discussion

The leptin–melanocortin pathway regulates energy expenditure and food intake. As the key regulators of the leptin–melanocortin pathway, MCRs and POMC-derived hormones are crucial for metabolism [70]. Loss-of-function mutations in the *POMC*, *MC4R*, and *MC3R* genes cause severe obesity, lowering the length and quality of life of the patients due to complications such as cardiovascular disease, type 2 diabetes, and NAFLD. Loss-of-function variants of POMC, MCRs, and other genes in the leptin–melanocortin pathway require broad therapeutic approaches. For a complete understanding of these mechanisms, the physical interactions between MCRs and their ligands, and the reaction kinetics and genetic regulation of these interactions, must be investigated. To this end, the recently discovered second promoter of *POMC* and the novel variant in the KKRRP motif create new diagnostic and therapeutic research opportunities. For example, KRRP motif mutations could provide an opportunity for the development of simple and efficient PCR-based approaches similar to COVID-19 tests or quick enzyme-linked immunosorbent assays (ELISAs) for the diagnosis of genetic obesity [49,71].

When the phenotypes of patients with *MC3R* and *MC4R* mutations were compared, both genes were found to be involved in severe early-onset obesity. However, MC4R-related obesity was often associated with linear growth; fasting hyperinsulinemia; an increased risk of hypertension; reduced energy expenditure; and increases in lean mass, fat mass, and visceral fat. Interestingly, adult MC4R carriers are not at an increased risk of obesity-related complications such as diabetes but linked to cardiovascular dysfunctions [72]. On the other hand, MC3R carriers had the following phenotypic characteristics: an increased fat mass, decreased lean mass, no change in energy expenditure, and delayed sexual maturity. Moreover, MC3R was found to be crucial for the activation of AgRP neurons during fasting, cold exposure, or ghrelin stimulation [73]. However, AgRP was found to inhibit both the MC3R and MC4R G_s_α coupling pathways while activating the extracellular signal-regulated kinase 1/2 (ERK1/2) pathway. Also, MC4R was related with the activation of G_i_α, Akt (protein kinase B), and KiR 7.1 (potassium channel) with AgRP stimulation [72]. Investigations into the molecular mechanisms of loss-of-function mutations in MC4R revealed defects in ligand interactions, membrane expression, and intracellular transport, as well as constitutive activity. The underlying cellular and molecular mechanisms of MC3R mutations have not been fully explored [72,74,75]. Mutant MC3R underlying mechanisms are limited to G_s_α coupling-related cAMP levels [72,75]. More detailed cellular mechanism investigations into MC3R mutations are required to compare the mechanisms underlying the different MC3R mutant phenotypes. 

Ong et al. [62] found that the loss of function of MC3R was unrelated to puberty, while Lam et al. [41] found this variant to be related to the late pubertal stage and an increased estrous cycle length. Similarly to MC3R, which also controls the circadian rhythm, recent studies have revealed that POMC is also involved in growth and development. Wan et al. [76] indicated that POMC affects sexual dimorphism and energy balance in tilapia due to its high estrogen levels, which upregulate the expression of POMC, leading to feeding suppression and slower growth [76]. Sexually dimorphic phenotype variations due to POMC mutations indicate a need for sex-specific investigations in both animals and humans. Unfortunately, sexual dimorphism studies relating to MCR and POMC loss-of-function phenotypes have not yet produced clear results. The investigation of sexual dimorphism is crucial to understanding these phenotypes and developing effective therapeutic approaches.

The controversial results regarding loss-of-function variants of MC3R and their effects on pubertal stages could be due to the location of the point mutations and their structural consequences, such as their effect on protein–protein interactions due to changes in receptor conformation. For example, loss-of-function mutations of MC3R that are located at the interface of a protein interaction could impair the dimerization of the MC3R–GHSR1A complex. This heterodimer reduces GHSR1A-related activity and increases MC3R activity, further indicating the importance of molecular, structural, behavioral, and proteomics studies for MC3R [38]. In addition to MC3R dimerization or oligomerization with other GPCRs, MC3R and its coupling to G-proteins should be investigated, particularly with respect to G_q/11_α. The MC3R–GHSR1A heterodimer interaction was found to be a potential regulator of Gαs signaling from MC3R and the melanocortin pathway due to the fine tuning of the cAMP signal [34,36]. The literature suggests that, similarly to MC4R, MC3R may couple to a G_α_ subunit other than G_αs_ to control the appetite and energy expenditure pathways. A study on the loss-of-function MC3R Ala33Thr variant pointed towards obesity-related pathways controlled by G-proteins other than G_αs_, similar to MC4R [34]. Semaglutide, popularly known as “Ozempic^®^”, is a recently discovered antidiabetic drug that is prescribed to diabetic patients for weight control. Its mechanism involves the glucagon-like peptide 1 (GLP1) receptor, which was recently found to synergistically interact with MC3R [77]. MC3R-knockout mice that were administered the GLP1 agonist liraglutide showed enhanced sensitivity to liraglutide but, interestingly, did not develop sensitivity to incretins, which stimulate insulin secretion, indicating the antagonistic impact of MC3R on the GLP1 receptor [78]. Crystal structures of MC4R and MC3R recently released by Heyder et al. [69] and Feng et al. [66] further our understanding of the structural and biophysical characteristics of these receptors [66,69]. Therefore, more structural and protein interaction studies are required to further clarify the roles of POMC-derived neuropeptides, MC3R, and MC4R in genetic obesity.

## 5. Conclusions

In conclusion, POMC-derived neuropeptides, MC3R, and MC4R regulate the leptin–melanocortin pathway and modulate appetite, stress, and reward processes. Recent discoveries concerning their protein–protein interactions and potential biomarker regions have created new research and therapeutic study opportunities to address obesity and developmental disorders. Structural studies have broadened our understanding of these models and their implications.
